# Three New Species, New Records, and a Key to *Dryops* Olivier, 1791 (Coleoptera: Dryopidae) from Brazil

**DOI:** 10.3390/insects17040430

**Published:** 2026-04-17

**Authors:** Matheus de Souza Leite Alexandre, Bruno Clarkson, André Silva Fernandes, Felipe Ferraz Figueiredo Moreira

**Affiliations:** 1 Laboratório de Entomologia, Instituto Oswaldo Cruz, Fundação Oswaldo Cruz, Rio de Janeiro 21041-250, RJ, Brazil; matheusslalex@gmail.com (M.d.S.L.A.); ppmeiameiameia@gmail.com (F.F.F.M.); 2 Sistemática de Insetos, Laboratório de Entomologia e Fitopatologia, Universidade Estadual do Norte Fluminense Darcy Ribeiro, Campos dos Goytacazes 28013-602, RJ, Brazil; 3 Laboratório de Entomologia, Coordenação do Curso de Ciências Biológicas, Universidade Federal do Tocantins, Porto Nacional 77500-000, TO, Brazil; andresf@uft.edu.br

**Keywords:** aquatic beetles, biodiversity, Dryopoidea, Elateriformia, morphology

## Abstract

Dryopidae (Insecta: Coleoptera) comprise 37 genera and about 280 species, occurring on all continents except Antarctica. The Neotropical fauna of the family has been neglected for decades, despite a few recent contributions. So far, seven genera and 29 species have been recorded from Brazil. Based on 369 adults collected in the Brazilian states of Bahia, Paraná, and Rio de Janeiro, we describe *Dryops antonioi* **sp. nov.**, *D. nelsimarae* **sp. nov.**, and *D. simoneae* **sp. nov.** Furthermore, we herein redescribe and provide new records for *D. ovatus* (Grouvelle, 1890), an updated key to the species recorded from the country and a checklist of Neotropical representatives of the genus.

## Simple Summary

Beetles are the most diverse group in terms of number of species, but the majority of their biodiversity remains poorly known. This is true to Dryopidae beetles, which occurs on all continents except Antarctica. This study aims to contribute to the knowledge about Brazilian beetle fauna. Here we studied 369 adults collected in the Brazilian states of Bahia, Paraná, and Rio de Janeiro, identified as four different species of the genus *Dryops* Olivier, 1791. Three of them are new species, described here as *Dryops antonioi* **sp. nov.**, *D. nelsimarae* **sp. nov.**, and *D. simoneae* **sp. nov.** We also redescribe and provide new occurrence records for *Dryops ovatus* (Grouvelle, 1890) and an identification key to the species recorded from the country.

## 1. Introduction

With about 386,000 described species, beetles (Insecta: Coleoptera) represent the most diverse animal group. Dryopidae (Coleoptera: Elateriformia: Dryopoidea) comprise 37 genera and about 280 species and occur all over the planet, except in Antarctica (Bouchard et al. 2017 [1] (pp. 337–417); Jäch & Balke, 2008 [2] (pp. 419–442); Bilton & Shepard, 2022 [3] (pp. 539–553); Polizei et al., 2022 [4] (pp. 256–269)). Most of the known larvae (*Dryops* Olivier, 1791 [5], *Helichus* Erichson, 1847 [6] (pp. 67–185), *Pelonomus* Erichson, 1847 [6] (pp. 67–185), *Pomatinus* Sturm, 1853 [7], and *Postelichus* Nelson, 1989 [8] (pp. 19–24)) are terrestrial, and pupation occurs out of the water. Aquatic adults are not able to swim, so they are found crawling on live vegetation or decaying vegetal substrates in or out of the water or on gravel and rocks in streams. Riparian species can be found above or below the water along stream margins. There are also terrestrial species in which the adults are found on forest leaf litter, on flooded debris, or even on the foliage of trees and plants (like in arboreal groups, such as some *Sostea* Pascoe, 1860 [9] (pp. 36–64)). Regardless of the habitat, they all can be collected using light traps at night (Thorp & Rogers, 2015 [10]; Kodada et al., 2016 [11] (pp. 590–602)). The Neotropical fauna of Dryopidae includes 15 genera and 68 valid species, of which the following ten genera are aquatic or semiaquatic: *Dryops*; *Elmoparnus* Sharp, 1882 [12] (pp. 119–140); *Guaranius* Spangler, 1991 [13] (pp. 147–151); *Helichus* Erichson, 1847 [6] (pp. 67–185); *Microparnus* Shepard, 2019 [14] (pp. 62–66); *Novopelmus* Shepard, 2020 [15] (pp. 99–128); *Onopelmus* Spangler, 1980 [16] (pp. 31–35); *Parygrus* Erichson, 1847 [6] (pp. 67–185); *Pelonomus*; and *Platyparnus* Shepard and Barr, 2018 [17] (pp. 209–226) (Shepard & Barr, 2018 [17] (pp. 209–226); Kodada et al., 2016 [11] (pp. 590–602); Polizei et al., 2022 [4] (pp. 256–269)). In Brazil, there are records of 29 species belonging to seven genera (*Dryops*, *Helichus*, *Onopelmus*, *Parygrus*, *Pelonomus*, *Platyparnus,* and *Sostea*) (Sampaio et al., 2025 [18]). The study of the family in Brazil and the Neotropics as a whole has been neglected for decades, despite a few recent contributions (e.g., Shepard & Barr, 2018 [17] (pp. 209–226); Shepard, 2019 [14] (pp. 62–66), 2020 [15] (pp. 619–625); Barr & Shepard, 2020 [19] (pp. 99–128); Polizei et al., 2022 [4] (pp. 256–269)). Among the Neotropical genera of the family, *Dryops* is the most problematic because of the generally poor descriptions and the scarcity of revisionary studies and illustrations. This genus comprises about 79 species and is considered cosmopolitan (Shepard & Sites, 2016 [20]). Based on 369 adults collected in the Brazilian states of Bahia, Paraná, and Rio de Janeiro, we herein describe *Dryops antonioi* **sp. nov.**, *D. nelsimarae* **sp. nov.**, and *D. simoneae* **sp. nov.** Furthermore, we redescribe and provide new records for *D. ovatus* (Grouvelle, 1890 [21] (pp. 155–158)), as well as an updated key to the species recorded from the country and a checklist (Table S1) of Neotropical representatives of the genus.

## 2. Material and Methods

**Figure 1:**
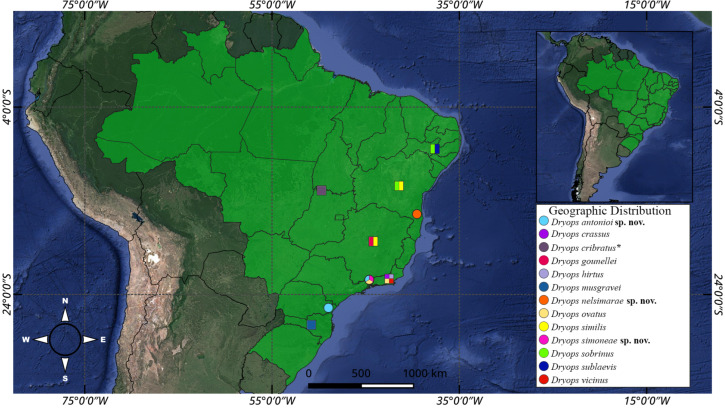
Map of geographic distribution of the Brazilian *Dryops* species. Circles = records from this study. Squares = records in literature. Asterisk = Brazil is the only known locality.

The material examined was collected in the Parque Nacional (PARNA) do Itatiaia and the PARNA da Serra dos Órgãos (Rio de Janeiro) in the Parque Estadual Pico do Marumbi (Paraná) and in the municipality of Camacan (Bahia) (Figure 1; Table S2). Except for Camacan, all these localities are at 900–1200 m a.s.l., with a subtropical highland climate. Camacan lies at 160 m a.s.l., with a tropical humid climate (Guimarães & Arlé, 1984 [22] (pp. 309–323); Mota et al., 2009 [23] (pp. 747–770); Richter, 2013 [24] (pp. 91–100); Vashchenko & Biondi, 2013 [25] (pp. 108–118)).

Part of the beetles were collected in both lotic and lentic habitats by dragging an aquatic D-net close to the marginal vegetation. Other specimens were collected using light traps (white sheet) near water bodies, and one was found on leaf litter out of the water in the riparian zone. Specimens were preserved in 75% ethanol, then transferred to 90% ethanol and kept in freezers at −20 °C. Some were pinned posteriorly.

Identification at genus level was performed by following Shepard (2020 [15] (pp. 99–128)). Species were identified based on original descriptions of Grouvelle (1890 [21] (pp. 155–158), 1896 [26] (pp. 33–52)), Hinton (1937 [27] (pp. 6–12)) and by comparison with photographs of type-specimens.

The general morphological terminology follows Lawrence (2011 [28] (pp. 1–217)). Total body length corresponds to the sum of the lengths of the pronotum and the elytron, excluding the head and the variable space between the pronotum and the elytra. Maximum width was measured through both elytra at their widest point (approximately at the apical 3/4), while maximum height was measured in lateral view at about 1/3 basal elytra and maximum pronotal width was measured at about 1/3 apical. For measurements on descriptions, average values among the specimens examined were considered. Male and female genitalia were detached from the specimens and cleaned in warm 10% KOH (Brown, 1972 [29]). They were observed, photographed on excavated microscope slides, stored in microvials with glycerin, and kept in the same pin as the specimen. Genitalic measurements were taken in lateral view.

A Zeiss Stemi SV6 stereoscope was used for morphological studies. Measurements were taken with the aid of a calibrated micrometer attached to the same stereoscope. Photographs were taken with a LEICA DMC2900 and the LEICA Application Suite V4.7, then edited with Adobe Photoshop CC and Adobe Lightroom CC. Maps were produced with QGIS (3.28LTR).

Labels are quoted verbatim. Changes of lines are indicated by “/”, different labels are separated by “|”, and labels of the same series end in “;”. Most specimens we collected are deposited in CEIOC—Coleção Entomológica do Instituto Oswaldo Cruz, Fundação Oswaldo Cruz, Rio de Janeiro, Brazil. Some of the specimens of *Dryops nelsimarae* **sp. nov.** are deposited in the following collections (12 paratypes in each):

CEUFT—Coleção de Entomologia da Universidade Federal do Tocantins, Laboratório de Entomologia, Porto Nacional, Brazil.
MEMH—Museu de Entomologia Magali Hoffmann da Universidade Estadual do Norte Fluminense Darcy Ribeiro, Campos dos Goytacazes, Brazil.
MZUSP—Museu de Zoologia da Universidade de São Paulo, São Paulo, Brazil.
Photographs provided by the Muséum National d’Histoire Naturelle, Paris, France, were also studied. The labels are as listed below.
*Dryops crassus* Grouvelle, 1896: crassus/Grouv.|Parnus/crassus/A. Grouv|Type|crassus of Brasil.
*Dryops cribratus* Grouvelle, 1896: Brasil|Type|Parnus/cribratus/A. Grouv.
*Dryops gounellei* Grouvelle, 1896: Type|Caraça (Minas Geraez)/Brésil/EGounelle 12.1885|gounellei/Grouv.
*Dryops hirtus* Grouvelle, 1896: Rio d. Jane/Brésil.|Type|Parnus/hirtus/A. Grouv.; hirtus/Grouv.
*Dryops ovatus* (Grouvelle, 1890): Bresil/Theresópolis|Type|Parnus/ovatus/A. Grouv.
*Dryops similis* Grouvelle, 1896: similis/Grouv.|Caraça (Minas Geraez)/Brésil/EGounelle 12.1885|Type.
*Dryops sobrinus* Grouvelle, 1896: sobrinus/Grouv.|Pery-Pery/(Pernambuco)/Gounelle 11.12.1892|Type.
*Dryops sublaevis* Grouvelle, 1896: Pery-Pery/(Pernambuco)/Gounelle 11.12.1892|Parnus/sublaevis/Grouv.|Type.
*Dryops vicinus* Grouvelle, 1896: vicinus/Grouv.|Type|Bresil/Theresópolis|Parnus/vicinus/A. Grouv.

A literature search for geographic distribution of *Dryops* species was conducted and the checklist of Neotropical *Dryops* (Table 1) was made following Blackwelder (1945 [30] (pp. 202–249)), Boukal et al. (2012 [31] (pp. 92–104)), Brown (1975 [32] (pp. 149–160)), Buczyński & Przewoźny (2008 [33] (pp. 199–215)), Darlington (1936 [34] (pp. 39–51)), de Taşar (2018 [35] (pp. 1–6)), Olmi (1972 [36] (pp. 69–132)), Garrido & Gayoso (2005 [37] (pp. 248–260)), Jach & Avtzis (1998 [38] (pp. 112–126)), Kodada & Merkl (1996 [39] (pp. 50–65)), Sanderson (1953 [40] (pp. 148–163)), Boukal et al. (2012 [41] (pp. 1–21)), Rico (1998 [42] (pp. 53–59)), Mascagni & Meloni (2011 [43] (pp. 135–142)), Prokin et al. (2017 [44] (pp. 239–240)), Przewoźny & Grabowski (2010 [45] (pp. 113–125)), El-Hassan (2023 [46] (pp. 101–116)), Benamar et al. (2022 [47] (pp. 335–389)), Salah (2017 [48] (pp. 32–40)), Satô (1979 [49] (pp. 56–68)), Shapovalov et al. (2012 [50] (pp. 211–212)), Shepard & Aguilar (2010 [51] (pp. 88–100)), Shepard (2004 [52] (pp. 130–145)), Shepard & Chaboo (2015 [53] (pp. 75–90)), Webster & DeMerchant (2012 [54] (pp. 80–92)) and Yoshitomi & Haga (2018 [55] (pp. 45–59)).

## 3. Results

**Taxonomy**

***Dryops* Olivier, 1791**

**Diagnosis.** Body dorsally pubescent or with tomentum, lateral margins of elytra without densely pubescent depression, and pronotum with complete sublateral/carina on each side.

**Descriptions**

**Figure 2:**
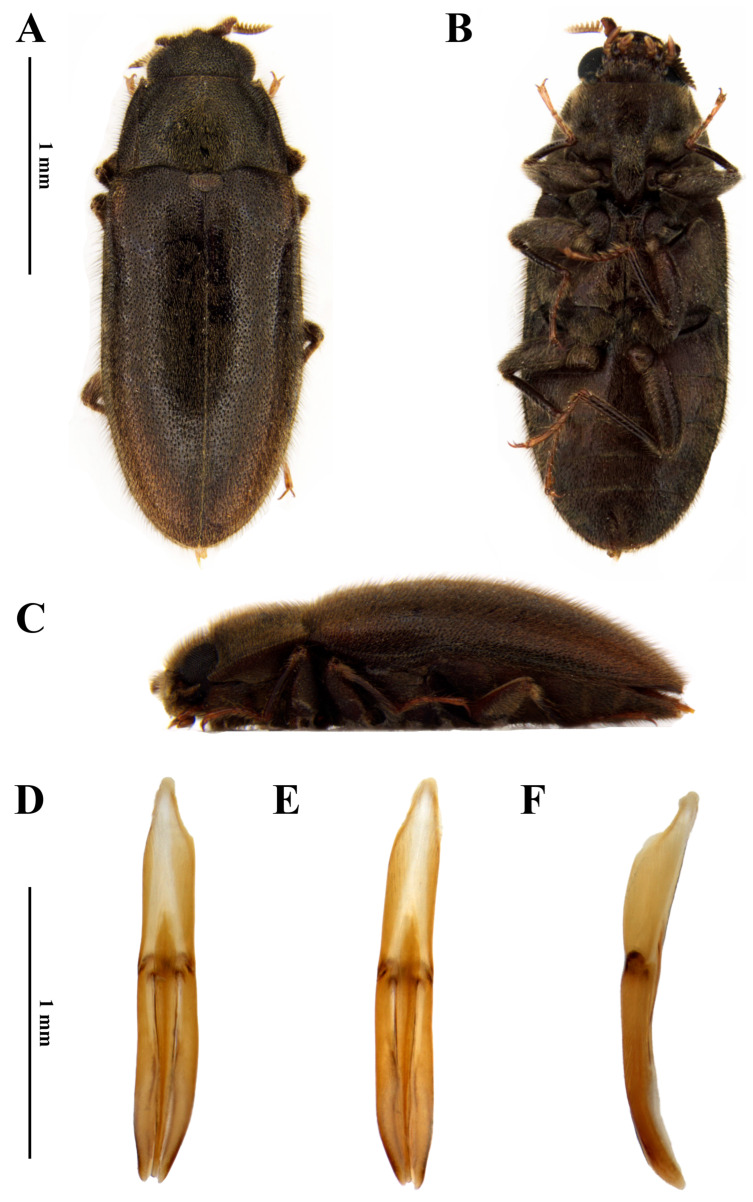
*Dryops nelsimarae*. Male holotype. *Habitus* and genitalia. (**A**) Dorsal view; (**B**) ventral view; (**C**) lateral view; (**D**) dorsal view; (**E**) ventral view; (**F**) lateral view.

**Figure 3:**
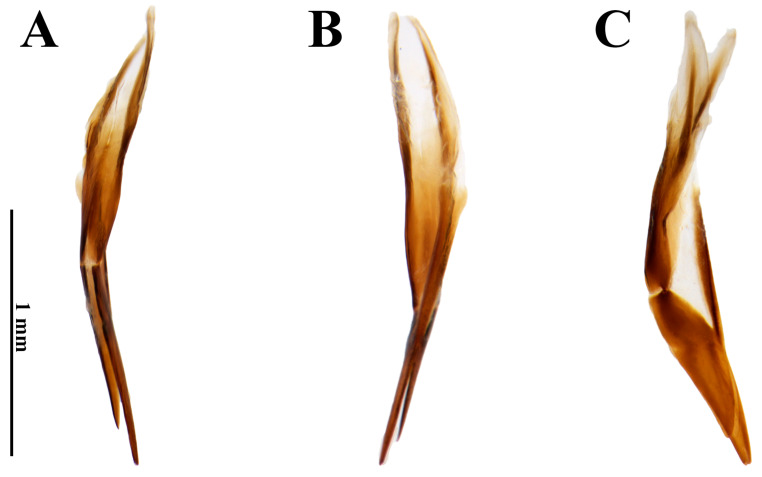
*Dryops nelsimarae*. Female genitalia. (**A**) Dorsal view; (**B**) ventral view; (**C**) lateral view.

***Dryops nelsimarae* sp. nov.** (Figure 2 and Figure 3)

urn:lsid:zoobank.org:act:15627D5E-6E89-4E07-9E18-9E238C4BED5D

**Type locality.** Camacan, Bahia state, BRAZIL.

**Male holotype:** CEIOC 83064|Brasil: BA, Camacan,/BA-662, ponte sobre o Rio/Panelão, 5.IV.2023|Luz, pano branco/15°25′18.70″ S/39°31′43.70″ W, 161 m/B. Clarkson col.

**Paratypes (73):** (11) CEIOC 83065|Brasil: BA, Camacan,/BA-662, ponte sobre o Rio/Panelão, 5.IV.2023|Luz, pano branco/15°25′18.70″ S/39°31′43.70″ W, 161 m/B. Clarkson col.; (8) CEIOC 83079|same data; (10) CEIOC 83080|same data; (8) CEIOC 83081|same data; (12) CEUFT, (12) MEMH, and (12) MZUSP|same data.

**Diagnosis.** Among Brazilian *Dryops*, *D. nelsimarae* **sp. nov.** is only similar to *D. sublaevis* due to the more elongated and narrowed body, but it is shorter, about 4.5 mm long, while *D. sublaevis* is about 6 mm long. Other characteristics that distinguish these species are the more evident elytral constriction and the more prominent humeral region in *D. nelsimarae* **sp. nov.**

**Description**

*Size and shape*. *Male* (Figure 2A–C). Total length 4.3–4.5 mm (n = 5); maximum width 2 mm; maximum head width 1.37 mm; elytral length 3.3 mm; maximum pronotal width 1.7 mm; maximum height 1.5 mm. Body oblong, moderately convex, densely covered by setae and punctations which are evenly distributed on dorsum and venter.

*Coloration*. Dorsum of head, pronotum, scutellum, and elytra uniformly dark brown, covered with golden setae on head and elytra, with brown and golden setae on pronotum. Venter dark reddish brown. Anterior and posterior margins of prosternum, mesoventrite and metaventrite paler. Antenna dark brown. Maxillary and labial palpi brown. Terminal palpomere of maxillary palpi darker; terminal palpomere of labial palpi paller at apical third. Legs dark brown to reddish brown. Apex of tibia and tarsus progressively paler.

*Head*. Labrum setose, punctate, with anteromedial portion glabrous; anterior margin sinuous. Clypeus truncate at anterior margin; surface setose, punctate. Antenna with 11 antennomeres; scape and pedicel covered by long setae; antennomeres 3–11 densely covered by short setae; scape thickened; pedicel strongly thickened, covering antennomeres 3–5 in dorsal view. Maxillary palpi 4-articulated; IV > I > II > III; palpomeres I–III setose; I with posterior margin curved laterally; II thickening towards apex, posterior margin straight, slightly curved mesally; III discreetly thickening towards apex, posterior margin straight, discreetly curved mesally; IV without setae, strongly thickened, slightly longer than I–III together, apical half narrowing towards apex, apex strongly sharp. Labial palpi shorter than maxillary palpi, 4-articulated; IV > II > I = III; palpomeres I–III setose; I thinnest, short; II elongated, straight; III short, apex beveled, strongly curved mesally; IV slightly longer than II, slightly flattened, robust, apex rounded.

*Prothorax*. Pronotum convex, densely punctate; punctations as intense as on head; with moderately long, erect setae; anterior margin straight, except at strongly acute, depressed, anterolateral angles. Prosternum forming a longitudinal plate on anterior portion, forming a “chin-piece” that receives head in resting position and covering it when retracted; prosternal process projected posteriorly between procoxae; procoxae extending to the anterior margin of mesoventrite.

*Pterothorax*. Scutellar shield wider than long, subtriangular, very convex laterally. Elytron punctate; punctations as dense as on head and pronotum; surface covered with moderately long leaning setae; punctations forming striae only in lighter specimens (teneral adults). Humeral region prominent, protruding beyond contour of elytral disc due to a constriction posterior to humeral region. Elytra together much longer than wide. Hindwing macropterous. Metaventrite reduced to two subtriangular plates, with distal-most portion forked into two acute points.

*Legs*. Covered with punctations and long, erect, golden setae; setae forming a row of combs on mesal side of femora. Femora with a posterior depressed process forming a groove to receive the tibia. Tibia with well-spaced setae; pro- and mesotibia tibia weakly arcuate, metatibia straight. Each tarsus with five tarsomeres with long, erect setae on ventral face, except on distal tarsomere, which is about as long as the three preceding ones combined, with two regular apical claws without basal or subbasal teeth.

*Abdomen*. Ventrite 1 with triangular intercoxal process projected, slightly depressed between metacoxae; ventrites 2–4 similar to each other; ventrite 5 longest.

*Genitalia*. *Aedeagus* (Figure 2D–F). Total length 1.5 mm. Phallobase slightly shorter than parameres; asymmetrical at base. Parameres slightly surpassing apex of median lobe, curved ventrally at apex; mesal margin straight; thicker than median lobe, especially at apical half; apex acuminate. Median lobe with about 1 mm; lanceolate in dorsal and ventral views, slightly falcate in lateral view, curved ventrally at apex; fibula long, uniform in diameter; occupying less than half of the phallobase and almost reaching apices of parameres.

*Female*. *Size and shape*. Total length 4.4–4.5 mm (n = 5); maximum width 2.1 mm; maximum head width 1.37 mm; elytral length 3.65 mm; maximum pronotal width 1.9 mm; maximum height 1.6 mm. External morphology similar to males. *Ovipositor* (Figure 3). Total length 1.85 mm. Paraprocts asymmetrical. In dorsal view, right paraproct convex, left paraproct sinuous; slightly more sclerotized than proctiger; middle portion sharp and heavily sclerotized, extending longitudinally up to apices; less sclerotized lateral portion on both sides at approximately apical 2/3 up to the apices. In dorsal view, apices of paraprocts split at approximately apical 1/5 and proctiger completely split into asymmetrical right and left gonocoxites. Left gonocoxite with approximately 3/4 of right gonocoxite length. Gonocoxites ventrally fused only at base of baculi. Baculi with about ⅘ the length of right gonocoxite.

*Geographic distribution* (Figure 1). Known only from the type locality in the state of Bahia, Brazil.

*Etymology.* This species is named in honor of the senior author’s grandmother, Nelsimar de Souza Leite.

*Habitat notes.* We collected 344 adults during a single night in April (autumn), by using a white sheet light trap. This may indicate a reproductive or dispersal period of this species around that season (Boda & Csabai 2013 [56] (pp. 133–147)).

**Figure 4:**
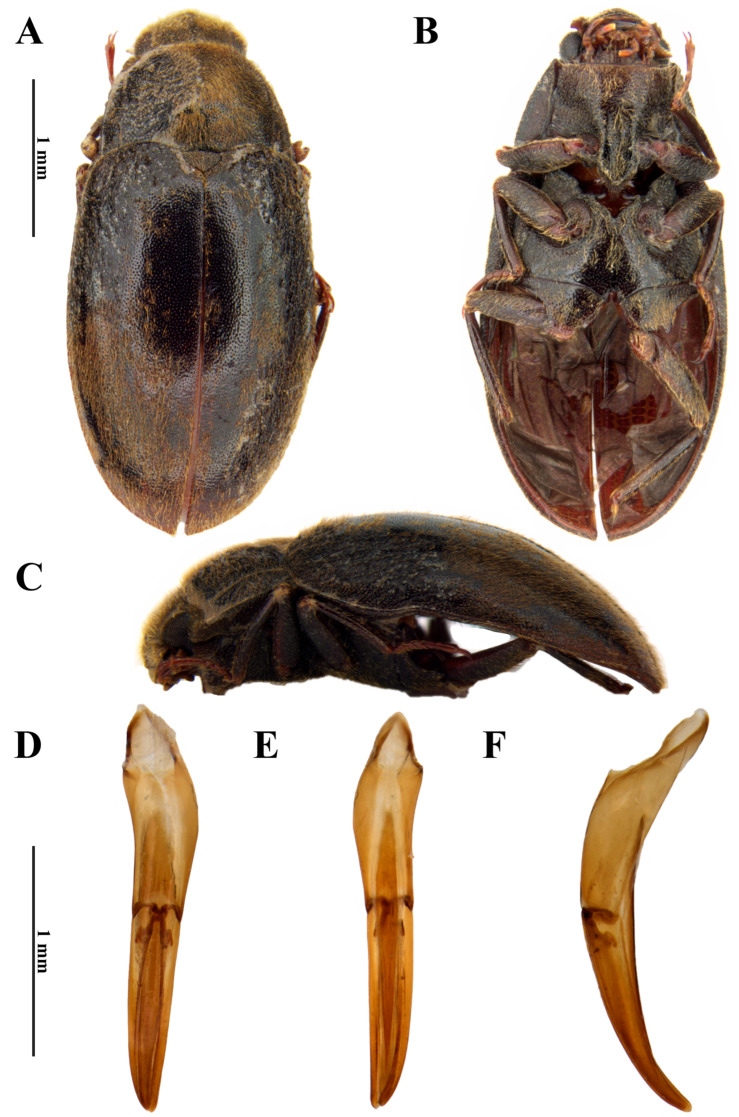
*Dryops antonioi*. Male holotype. *Habitus* and genitalia. (**A**) Dorsal view; (**B**) ventral view; (**C**) lateral view; (**D**) dorsal view; (**E**) ventral view; (**F**) lateral view.

**Figure 5:**
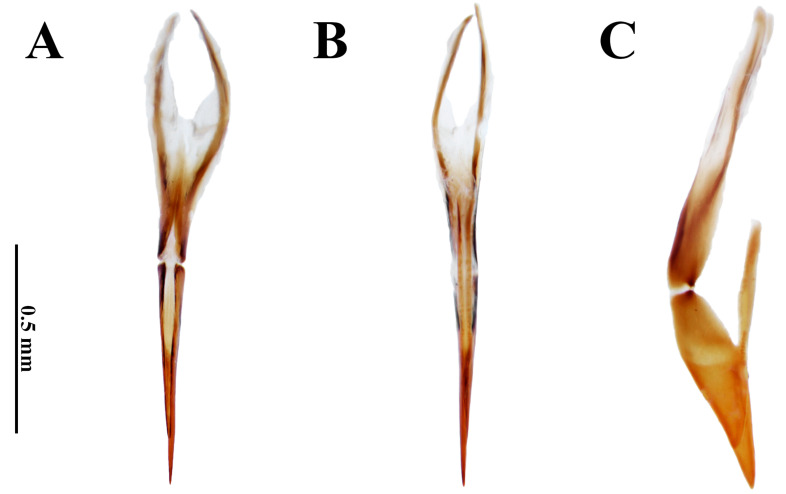
*Dryops antonioi*. Female genitalia. (**A**) Dorsal view; (**B**) ventral view; (**C**) lateral view.

***Dryops antonioi* sp. nov.** (Figure 4 and Figure 5)

urn:lsid:zoobank.org:act:5F4A8757-A48F-4BCC-90FD-3C9D32A201C1

**Type locality.** Itatiaia, state of Rio de Janeiro, BRAZIL.

**Male holotype:** CEIOC 83062|Brasil: RJ, Itatiaia, PNI,/córrego na trilha Ruy/Braga, Cachoeira Véu da/Noiva, 18.XII.2021|22°25′38″ S 44°37’07″ W/1179 m/B. Clarkson col.

**Paratypes(5):** Paraná state: CEIOC 83078|Brasil: Paraná, Piraquara, Parque Estadual/Pico do Marumbi, 20.IX.2019, Mananciais da Serra,/901m, 25°25’47″ S 48°58’51″ W,/ B. Clarkson col. Rio de Janeiro state: CEIOC 83063|Brasil: RJ, Itatiaia, PNI,/riacho na trilha para/Cachoeira Poranga,/19.XII.2021|22°26’00″ S 44°37’00″ W/1062 m/ B. Clarkson col.; CEIOC 83077|Brasil: Rio de Janeiro, Itatiaia, PARNA de Itatiaia,/19.XII.2021, riacho na trilha para Cachoeira/Poranga, 1062 m, 22°26’00″ S 44°37’00″ W,/ B. Clarkson col.; CEIOC 83076|Brasil: Rio de Janeiro, Itatiaia, PARNA de Itatiaia, 18.XII.2021,/córrego na trilha Ruy Braga, Cachoeira Véu da Noiva,/1179 m, 22°25’38″ S 44°37’07″ W,/ B. Clarkson col.

**Diagnosis.** Among Brazilian *Dryops*, *D. antonioi* **sp. nov.** is only similar to *D. ovatus* and *D. simoneae* **sp. nov.** (*ovatus* species group) because of the strongly oval body, but it can be distinguished from them by the 5.9–6.2 mm total length (5.7–6.0 mm in *D. ovatus*; 5.4–5.7 mm in *D. simoneae* **sp. nov.**); the male phallobase, in dorsal view, with the same size as the parameres (slightly longer in *D. ovatus*; slightly shorter in *D. simoneae* **sp. nov.**); and the female baculi with a little more than half the length of the gonocoxites (slightly shorter in *D. ovatus*; much shorter in *D. simoneae* **sp. nov.**).

**Description**

*Size and shape*. *Male* (Figure 4A–C). Total length 6–6.2 mm (n = 2); maximum width 2.6 mm; maximum head width 1.37 mm; elytral length 4 mm; maximum pronotal width 2.5 mm; maximum height 2.3 mm. Body strongly oval, moderately convex, densely and uniformly covered by setae and punctations.

*Coloration*. Dorsum of head, pronotum, scutellum, and elytra uniformly dark brown, covered with golden setae on head and elytra, with brown and golden setae on pronotum. Venter dark brown. Antenna dark brown. Maxillary and labial palpi brown. Terminal palpomere of maxilla darker. Labial palpomeres darker at basal half. Legs dark brown to reddish brown. Trochanters, anterior margin of femora, posterior end of tibia, and tarsi paler.

*Head*. Labrum setose, punctate; with an anteromedial portion glabrous; anterior margin sinuous. Clypeus nearly truncate at anterior margin; surface setose, punctate. Antenna with 11 antennomeres; scape and pedicel covered by long setae; antennomeres 3–11 densely covered by short setae; scape thickened; pedicel strongly thickened, covering antennomeres 3–5 in dorsal view. Maxillary palpi 4-articulated; IV > I > II > III; palpomeres I–III setose; I with posterior margin curved laterally; II thickening towards apex, posterior margin straight, slightly curved mesally; III discreetly thickening towards apex, posterior margin straight, discreetly curved mesally; IV without setae, strongly thickened, slightly longer than palpomeres I–III together, apex narrowing towards apex, beveled at lateral margin, rounded at posterior margin, straight at mesal margin. Labial palpi shorter than maxillary palpi, 4-articulated; IV > II > I = III; palpomeres I–III setose; I thinnest, short; II elongated, straight; III short, apex beveled, strongly curved mesally; IV slightly longer than II, slightly flattened, robust, apex rounded.

*Prothorax*. Pronotum convex; densely punctate, punctations as dense as on head; with moderately long, erect setae; anterior margin straight, except at strongly acute, depressed, anterolateral angles. Prosternum forming a longitudinal plate on anterior portion, forming a “chin-piece”, which receives head in resting position and covering it when retracted; prosternal process projected posteriorly between the procoxae, which extend to anterior margin of mesoventrite.

*Pterothorax*. Scutellar shield wider than long, subtriangular, very convex laterally. Elytron punctate; punctations as dense as on head and pronotum; surface covered with setae moderately long leaning setae; punctations not forming rows or striae. Without elytral constriction; humeral region not prominent. Elytra together longer than wide. Hindwing macropterous. Metaventrite reduced to two subtriangular plates, with distal-most portion forked into two acute points.

*Legs*. Punctation more spaced than on body; covered with long, erect golden setae; setae forming row of combs in mesal side of femora. Femora with posterior depressed process forming a groove to receive tibia. Tibia with well-spaced setae; pro- and mesotibia weakly arcuate, metatibia straight. Tibia with well-spaced setae; pro- and mesotibia tibia weakly arcuate. Each tarsus with five tarsomeres with long, erect setae on ventral face, except on distal tarsomere, which is about as long as the three preceding ones combined, with two regular apical claws without basal or subbasal teeth.

*Abdomen*. Ventrite 1 with triangular intercoxal process projected, slightly depressed between metacoxae; ventrites 2–4 similar to each other; ventrite 5 longest.

*Genitalia*. *Aedeagus* (Figure 4D–F). Total length 1.9 mm. Phallobase about as long as parameres; asymmetrical at base. Parameres reaching apex of median lobe, curved ventrally at apex; in dorsal view, mesal margin almost straight, widest at base; thinner than median lobe, especially at apical half; apex rounded. Median lobe with about 1.4 mm; lanceolate in dorsal and ventral views; slightly falcate in lateral view, slightly curved ventrally at apex; fibula long, basally wider, occupying a little more than half of phallobase, reaching apex of parameres.

*Female*. *Size and shape*. Total length 5.9–6.2 mm (n = 4); maximum width 3.12 mm; maximum head width 1.37 mm; elytral length 4.37 mm; maximum pronotal width 2.62 mm; maximum height 2.5 mm. External morphology similar to males. *Ovipositor* (Figure 5). Total length 1.27 mm. Paraprocts asymmetrical. In dorsal view, right paraproct convex, left paraproct sinuous; more sclerotized than proctiger; middle portion sharp and heavily sclerotized, extending longitudinally up to apices; less sclerotized lateral portion on both sides at approximately apical 2/3 up to the apices. In dorsal view, apices of paraprocts split at approximately basal 5⁄6 and proctiger completely split into asymmetrical right and left gonocoxites. Left gonocoxite with approximately 3/4 of right gonocoxite length. Gonocoxites ventrally fused and surrounding the vulva. Baculi with about ⅔ of right goconoxite.

*Geographic distribution* (Figure 1). Known from the states of Rio de Janeiro and Paraná, eastern Brazil.

*Etymology*. This species is named in honor of the senior author’s father, Antônio José Alexandre.

*Habitat notes*. Some specimens were found on leaf litter submerged in water and others on underwater vegetation in the stream.

**Figure 6:**
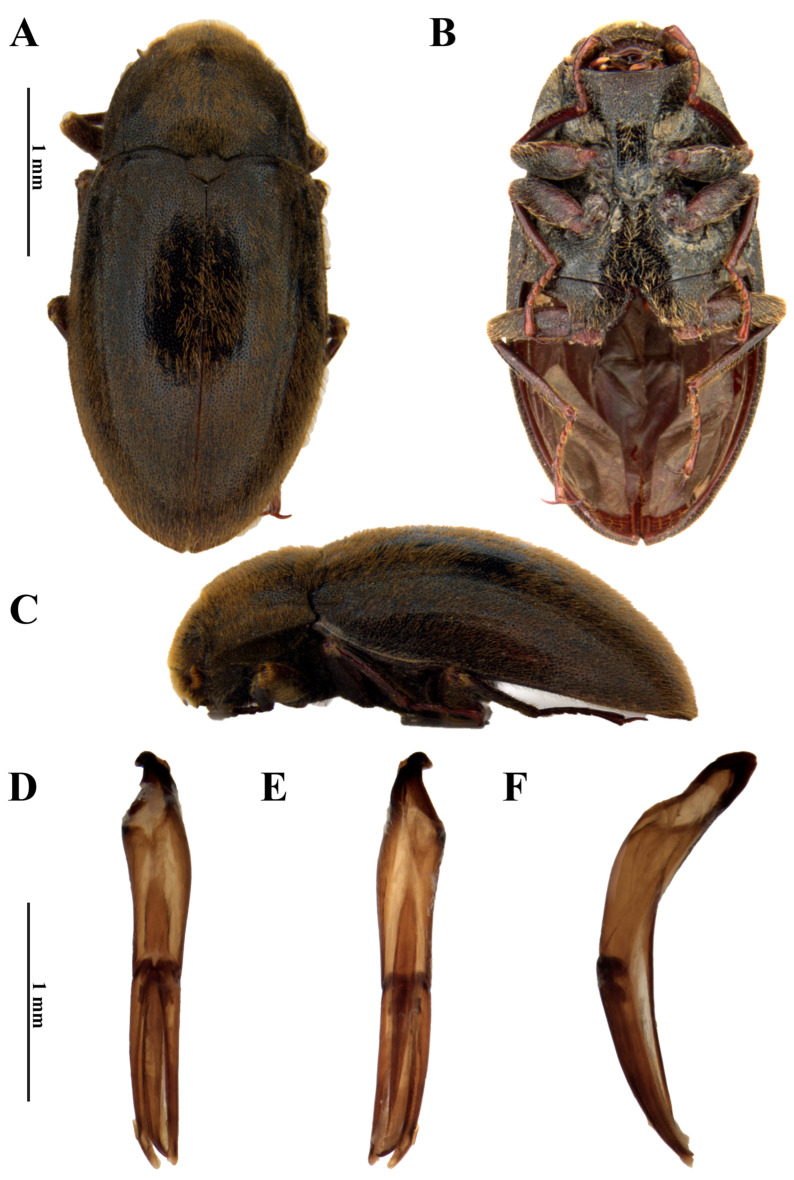
*Dryops ovatus*. Non-type. Male *habitus* and genitalia. (**A**) Dorsal view; (**B**) ventral view; (**C**) lateral view; (**D**) dorsal view; (**E**) ventral view; (**F**) lateral view.

**Figure 7:**
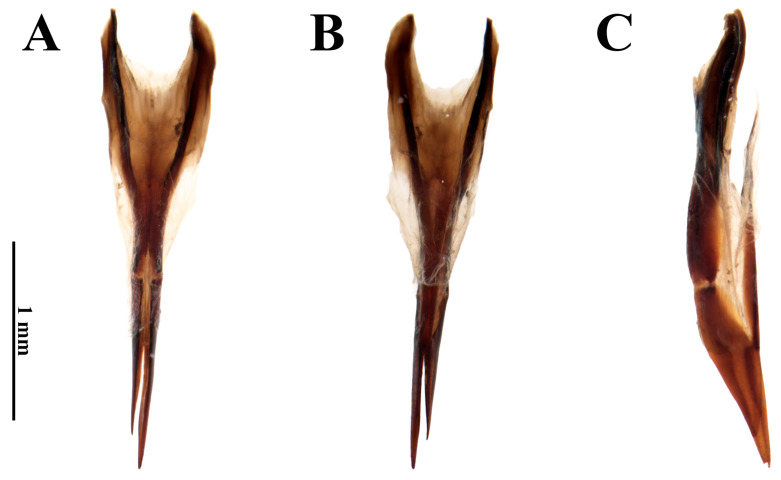
*Dryops ovatus*. Female genitalia. (**A**) Dorsal view; (**B**) ventral view; (**C**) lateral view.

***Dryops ovatus* (Grouvelle, 1890)** (Figure 6 and Figure 7)

*Parnus ovatus* Grouvelle, 1890: CXLVI.

*Dryops ovatus*: Grouvelle (1896: 18).

**Type locality.** Theresopolis (Brésil). See remarks.

**Material examined (11):** CEIOC 83068|Brasil: Rio de Janeiro, Itatiaia, PARNA de Itatiaia,/19.XII.2021, riacho na trilha para Cachoeira/Poranga, 1062 m, 22°26′00″ S 44°37′00″ W,/B. Clarkson col.; CEIOC 83069|same data; CEIOC 83082|same data; CEIOC 83083|same data.

**Diagnosis.** Among Brazilian *Dryops*, *D. ovatus* is only similar to *D. antonioi* **sp. nov.** and *D. simoneae* **sp. nov.** (*ovatus* species group). For a comparison, see diagnosis under *D. antonioi* **sp. nov.**

**Description**

*Size and shape*. *Male* (Figure 6A–C). Total length 5.7–6 (n = 7) mm; maximum width 3 mm; maximum head width 1.37 mm; elytral length 4.3 mm; maximum pronotal width 2.3 mm; maximum height 2.2 mm. Body strongly oval, moderately convex, densely covered by setae and punctations with the same density dorsally and ventrally.

*Coloration*. Dorsum of head, pronotum, scutellum, and elytra uniformly dark brown, covered with golden setae on head and elytra, with brown and golden setae on pronotum. Venter dark brown. Antenna with scape and pedicel dark brown; antennomeres 3–11 slightly paler. Maxillary and labial palpi brown. Terminal palpomere of maxillary and labial palpi paler distally. Legs dark brown to reddish brown. Tarsi paler.

*Head*. Labrum setose, punctate, with anteromedial portion glabrous; anterior margin sinuous. Clypeus nearly truncate at anterior margin; surface setose, punctate. Antenna with 11 antennomeres; scape and pedicel covered by long setae; antennomeres 3–11 densely covered by short setae; scape thickened; pedicel strongly thickened, covering antennomeres 3–5 in dorsal view. Maxillary palpi 4-articulated; IV > I > II > III; palpomeres I–III setose; I with posterior margin curved laterally; II thickening towards apex, posterior margin straight, slightly curved mesally; III discreetly thickening towards apex, posterior margin straight, discreetly curved mesally; IV without setae, strongly thickened, slightly longer than palpomeres I–III together, apex narrowing towards apex, beveled at lateral margin, rounded at posterior margin, straight at mesal margin. Labial palpi shorter than maxillary palpi, 4-articulated; IV > II > I = III; palpomeres I–III setose; I thinnest, short; II elongated, straight; III short, apex beveled, strongly curved mesally; IV slightly longer than II, slightly flattened, robust, apex rounded.

*Prothorax*. Pronotum convex; densely punctate, punctations as dense as on head; with moderately long, erect setae; anterior margin straight, except at strongly acute, depressed, anterolateral angles. Prosternum forming a longitudinal plate on anterior portion, forming a “chin-piece”, which receives head in resting position and covering it when retracted; prosternal process projected posteriorly between procoxae, which extend to anterior margin of mesoventrite.

*Pterothorax*. Scutellar shield wider than long, subtriangular, very convex laterally. Elytron punctate; punctations as dense as on head and pronotum; surface covered with moderately long leaning setae; punctations not forming rows or striae; without constrictions; humeral region not prominent. Elytra together longer than wide. Hindwing macropterous. Metaventrite reduced to two subtriangular plates, with distal-most portion forked into two acute points.

*Legs*. Covered with finer punctations than remaining of body and long, erect, golden setae; setae forming row of combs oriented to mesal side of femora. Femora with posterior depressed process forming a groove to receive the tibia. Tibia with well-spaced setae; pro- and mesotibia weakly arcuate, metatibia straight. Each tarsus with five tarsomeres with long, erect setae on ventral face, except on distal tarsomere, which is about as long as the three preceding ones combined, with two regular apical claws without basal or subbasal teeth.

*Abdomen*. Ventrite 1 with triangular intercoxal process projected, slightly depressed between metacoxae; ventrites 2–4 similar to each other; ventrite 5 longest.

*Genitalia*. *Aedeagus* (Figure 6D–F). Total length 2.1 mm. Phallobase a slightly longer than parameres; asymmetrical at base. Parameres extending beyond apex of penis, more weakly curved ventrally at apex than median lobe; in dorsal view, mesal margin straight, widest at acuminate apex; thinner than median lobe. Median lobe with 1.4 mm; lanceolate in dorsal and ventral views; slightly falcate in lateral view, weakly curved ventrally at apex; fibula long, slightly wider basally, occupying a little more than half of phallobase and reaching apex of parameres.

*Female*. *Size and shape*. Total length 6 mm (n = 1); maximum width 3.12 mm; maximum head width 1.37 mm; elytral length 4.5 mm; maximum pronotal width 2.50 mm; maximum height 2.25 mm. External morphology similar to males. *Ovipositor* (Figure 7). Total length 2.50 mm. Paraprocts symmetrical, slightly more sclerotized than proctiger, with a sharp, heavily sclerotized middle portion extending longitudinally up to apex; less sclerotized lateral portion on both sides ending at about basal 2/3. In dorsal view, apices of paraprocts split approximately at basal 2/3 and proctiger completely split into asymmetrical right and left gonocoxites. Left gonocoxite with about 4/5 of right gonocoxite total length. Gonocoxites ventrally fused only at the base of baculi. Baculi with about 9/10 the gonocoxites.

*Geographic distribution* (Figure 1). Ambiguous (see remarks). Collected in this study in a waterfall in the PARNA do Itatiaia, state of Rio de Janeiro, Brazil.

*Habitat notes*. The specimens were found in lotic habitats by dragging a D-net close to the marginal vegetation. The marginal vegetation was partially submerged in the water and the maximum depth was 1 m.

*Remarks*. The type locality is ambiguous. Grouvelle (1890 [21] (pp. 155–158)) cited “Theresopolis (Brésil)” in the original description of *D. ovatus*, which may refers to a locality in the municipality of Águas Mornas, Santa Catarina state (dos Passos et al. 2009 [57]), but in Grouvelle (1896 [26] (pp. 33–52)), the author cited “Hab. Brésil: Province de Rio de Janeiro—Collection Grouvelle”, which may indicate that the type locality is the municipality of Teresópolis, Rio de Janeiro state.

**Figure 8:**
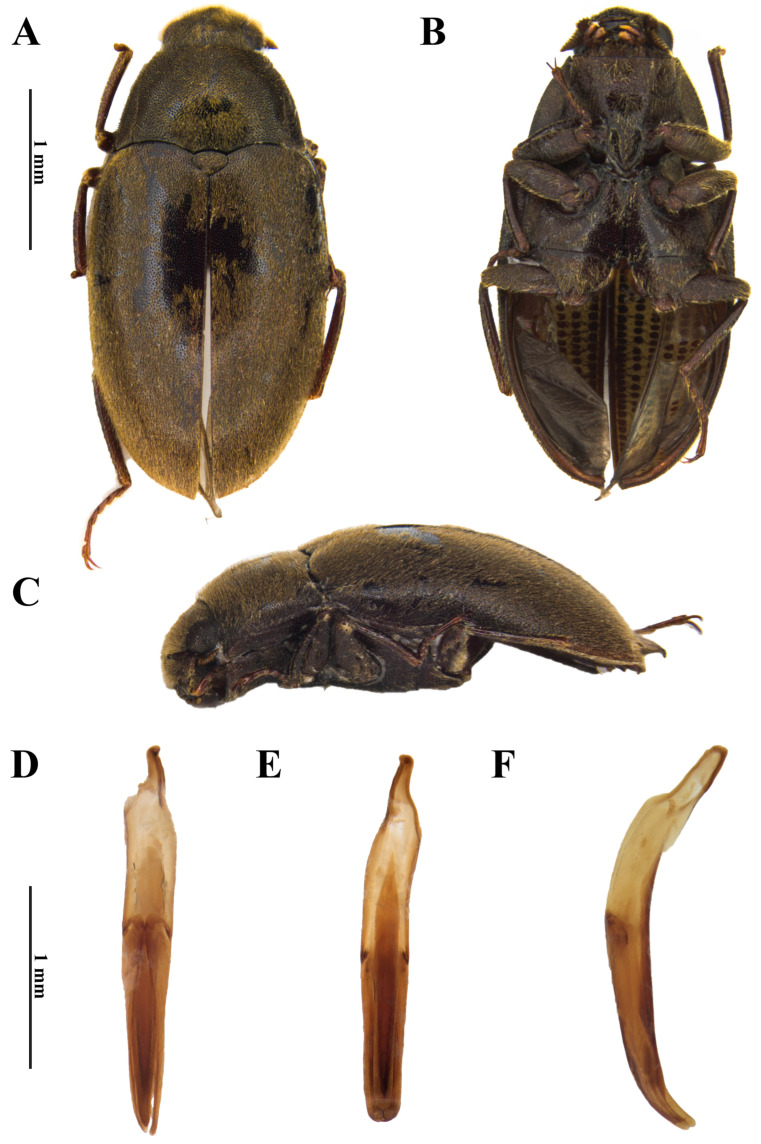
*Dryops simoneae*. Male holotype. *Habitus* and genitalia. (**A**) Dorsal view; (**B**) ventral view; (**C**) lateral view; (**D**) dorsal view; (**E**) ventral view; (**F**) lateral view.

**Figure 9:**
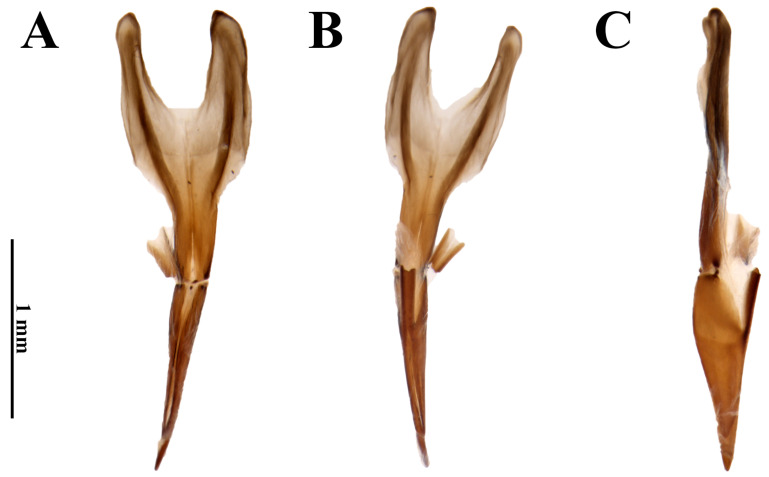
*Dryops simoneae*. Female genitalia. (**A**) Dorsal view; (**B**) ventral view; (**C**) lateral view.

***Dryops simoneae* sp. nov.** (Figure 8 and Figure 9)

urn:lsid:zoobank.org:act:950263B3-4532-4D0B-BF29-25B4DD7009A9

**Type locality.** Itatiaia, Rio de Janeiro State, BRAZIL.

**Male holotype:** CEIOC 83066|Brasil: RJ, Itatiaia, PNI,/riacho na trilha Vinícius de/Moraes, 18.XI.2021|22°27’22″ S 44°36’26″ W/795 m/B. Clarkson & M.S.L./Alexandre col.

**Paratypes(6):** CEIOC 83067| Brasil: RJ, Itatiaia, PNI,/riacho na trilha Vinícius de/Moraes, 18.XI.2021|22°27′22″ S 44°36′26″ W/795 m/B. Clarkson & M.S.L./Alexandre col.; CEIOC 83084|Brasil: Rio de Janeiro, Itatiaia, PARNA de Itatiaia,/18.XI.2021, riacho na trilha Vinícius de Moraes,/795 m, 22°27′22″ S 44°36′26″ W, B. Clarkson,/M.S.L. Alexandre col.; CEIOC 83085|same data; CEIOC 83086|same data.

**Diagnosis.** Among Brazilian *Dryops*, *D. simoneae* **sp. nov.** is only similar to *D. antonioi* **sp. nov.** and *D. ovatus* (*ovatus* species group). For a comparison, see diagnosis under *D. antonioi* **sp. nov.**

**Description**

*Size and shape*. *Male* (Figure 8A–C). Total length 5.4–5.6 mm (n = 4); maximum width 2.7 mm; maximum head width 1.37 mm; elytral length 3.75 mm; maximum pronotal width 2.3 mm; maximum height 2.0 mm. Body strongly oval, moderately convex, densely covered by setae and punctations with the same density dorsally and ventrally.

*Coloration*. Dorsum of head, pronotum, scutellum, and elytra uniformly dark brown, covered with golden setae on head and elytra, with brown and golden setae on pronotum. Venter dark brown. Antenna with scape and pedicel dark brown; antennomeres 3–11 paler. Maxillary and labial palpi brown. Terminal palpomere of maxilla paler at apical third. Terminal labial palpomere paler at apical half. Legs dark brown to reddish brown; tarsi brown.

*Head*. Labrum setose, punctate; anterior margin sinuous. Clypeus nearly truncate at anterior margin; surface setose, punctate; setae on anteromedial portion sparsely distributed. Antenna with 11 antennomeres; scape and pedicel sparsely setose; antennomeres 3–11 densely setose; scape thickened; pedicel strongly thickened, covering antennomeres 3–5 in dorsal view. Maxillary palpi 4-articulated; IV > I > II > III; palpomeres I–III setose; I with posterior margin curved laterally; II thickening towards apex, posterior margin straight, slightly curved mesally; III discreetly thickening towards apex, posterior margin straight, discreetly curved mesally; IV without setae, strongly thickened, slightly longer than palpomeres I–III together, apical half narrowing towards apex, apex beveled at lateral margin, rounded at posterior margin, straight at mesal margin. Labial palpi shorter than maxillary palpi, 4-articulated; IV > II > I = III; palpomeres I–III setose; I thinnest, short; II elongated, straight; III short, apex beveled, strongly curved mesally; IV slightly longer than II, slightly flattened, robust, apex rounded.

*Prothorax*. Pronotum convex; densely punctate, punctations as dense as on head; with moderately long, erect setae; anterior margin straight, except at strongly acute, depressed, anterolateral angles. Prosternum forming a longitudinal plate on the anterior portion, forming a “chin-piece”, which receives head in resting position and covering it when retracted; prosternal process projected posteriorly between procoxae, which extend to the anterior margin of the mesoventrite.

*Pterothorax*. Scutellar shield wider than long, subtriangular, very convex laterally. Elytra punctuated; punctations as dense as on head and pronotum; surface covered with moderately long leaning setae; punctations not forming rows or striae; without strangulation; humeral region not elevated. Elytra together longer than wide. Hindwing macropterous. Metaventrite reduced to two subtriangular plates, with distal-most portion forked into two acute points.

*Legs*. Punctations more spaced than on body; covered with long, erect, golden setae; setae forming a row of combs on mesal side of femora. Femora with a posterior depressed process forming a groove to receive the tibia. Tibia with well-spaced setae; protibia weakly arcuate; pro- and mesotibia arcuate, metatibia slightly curved. Each tarsus with five tarsomeres with long, erect setae on ventral face, except on distal tarsomere, which is about as long as the three preceding ones combined, with two regular apical claws without basal or subbasal teeth.

*Abdomen*. Ventrite 1 with triangular intercoxal process projected, slightly depressed between metacoxae; ventrites 2–4 similar to each other; ventrite 5 longest.

*Genitalia*. *Aedeagus* (Figure 8D–F). Total length 2.1 mm. Phallobase slightly shorter than parameres, asymmetrical at base. Parameres slightly longer than median lobe, curved ventrally at apex; mesal margin weakly sinuous, widest at base; much thinner than median lobe; apex rounded. Median lobe with about 1.5 mm; lanceolate in dorsal and ventral views; slightly falcate in lateral view, curved ventrally at apex; fibula long, basally wider; occupying more than half of phallobase, reaching apex of parameres.

*Female*. *Size and shape*. Total length 5.5–5.7 mm (n = 2); maximum width 2.8 mm; maximum head width 1.37 mm; elytral length 4.15 mm; maximum pronotal width 2.9 mm; maximum height 2 mm. External morphology similar to males. *Ovipositor* (Figure 9). Total length about 2.6 mm. Ovipositor slightly curved. Paraprocts symmetrical, sclerotized as proctiger, except more sclerotized basal 2/3; with a sharp, heavily sclerotized middle portion extending longitudinally up to apices. Lateral portions less sclerotized on both sides up to approximately basal 2/3. In dorsal view, apices of paraprocts split at about basal 2/3, and proctiger split into asymmetrical right and left gonocoxites. Left gonocoxite with about 4/5 of right gonocoxite total length. Gonocoxites ventrally fused only at the base of baculi. Baculi with about ½ gonocoxite.

*Geographic distribution* (Figure 1). Known from a few localities in the PARNA do Itatiaia, state of Rio de Janeiro, Brazil.

*Etymology*. This species is named in honor of the senior author’s mother, Simone de Souza Leite.

*Habitat notes*. This species was collected as those of *D. ovatus*, by dragging a D-net close to the marginal vegetation of lotic bodies of water. The marginal vegetation was partially submerged in the water and the maximum depth was 1 m.

**Key to adults of Dryops recorded from Brazil**

1. Total length (length of the pronotum plus the length of the elytron, excluding the head and the variable space between the pronotum and elytra) about 2.5 to 4 mm ............................................................................................................................................ 2
  Total length over 4 mm ................................................................................... 4
2. Elytra without lateral constriction .................................... *D. hirtus* Grouvelle, 1896
  Elytra with lateral constriction at basal 1/3 ............................................................ 3
3. Posterolateral angles of pronotum strongly acute. Margins of elytra not explanate ........................................................................ *D. similis* Grouvelle, 1896
  Posterolateral angles of pronotum almost right-angled. Margins of elytra slightly explanate ................................................................. *D. sobrinus* Grouvelle, 1896
4. Body strongly oval; elytra without lateral constriction ........... 5 (*ovatus* species group)
  Body oblong; elytra with lateral constriction .................................................................. 7
5. Total length about 4.8–6 mm; male phallobase, in dorsal view, slightly longer than paramere; female baculi slightly shorter than gonocoxite ........................................................................... *D. ovatus* (Grouvelle, 1890)
  Total length about 5.4–6.2 mm; male phallobase, in dorsal view, as long as paramere or slightly shorter; female baculi conspicuously shorter than gonocoxite (half or less of gonocoxite length) ..................................................................................... 6
6. Total length 5.9–6.2 mm; male phallobase, in dorsal view, as long as paramere; female baculi slightly longer than half of gonocoxite length ............................................................................... *D. antonioi* **sp. nov.**
  Total length 5.4–5.7 mm; male phallobase, in dorsal view, slightly shorter than paramere; female baculi much shorter than gonocoxite (less than half of gonocoxite length) .............................................................................. *D. simoneae* **sp. nov.**
7. Total length about 4–5 mm ............................................................................ 8
  Total length over 5 mm ................................................................................. 12
8. Elytra with well-marked punctations .............................................................. 9
  Elytra with thin, weakly marked punctations ................................................ 11
9. Pronotum with lateral margins explanate ........................ *D. crassus* Grouvelle, 1896
  Pronotum with lateral margins not explanate ................................................... 10
10. Total length about 4.9 mm; pronotum densely punctate, punctations similar to the ones on the head .......................................................... *D. musgravei* Hinton, 1937
  Total length about 4.5 mm; pronotum not densely punctate; head distinctly more deeply and densely punctate than pronotum .................. *D. gounellei* Grouvelle, 1896
11. Total length about 4.3–4.5 mm; elytra strongly constricted at basal 1/3; humeral region prominent ............................................................. *D. nelsimarae* **sp. nov.**
  Total length about 4.8 mm; elytral constriction very subtle; humeral region not prominent .......................................................... *D. vicinus* Grouvelle, 1896
12. Total length about 6.2 mm; elytra with thick punctations distributed in almost regular longitudinal lines; punctations not very dense ... *D. cribratus* Grouvelle, 1896
  Total length about 5.4–5.7 mm; elytra with thin punctations; punctations densely and irregularly distributed .......................................... *D. sublaevis* Grouvelle, 1896

## 4. Discussion

Here we propose the “*ovatus* species group” to encompass *Dryops antonioi* **sp. nov.**, *D. ovatus*, and *Dryops simoneae* **sp. nov.** due to their extremely similar external morphology. These three species share a noticeable very rounded body (strongly oval), whereas the remaining Brazilian Dryopidae have an oblong to subparallel body. The only way to clearly distinguish them consists in dissecting and observing the genitalia of both males and females.

Throughout the scarce literature on Neotropical Dryopidae, it is hard to find illustrations of the habitus and genitalia for the majority of the described species. The main authors that started working on the New World Dryopidae in the late 19th and early 20th centuries relied only on very generic textual descriptions and, in a few of them, poorly detailed line drawing illustrations for diagnostic features (e.g., Grouvelle, 1890 [21] (pp. 155–158); Hinton, (1937 [27] (pp. 6–12))). In recent years, we have seen increasingly effort toward updating our taxonomic knowledge on Neotropical Dryopidae, including updated generic definitions (Barr & Shepard, 2020 [19] (pp. 99–128); Shepard & Barr, 2018 [17] (pp. 209–226)) that made it possible for us and other research groups to also begin to make contributions to the family taxonomy. However, we are still far from close to solving the major problems like systematics and revision of the most specious genera. We also reinforce the taxonomic potential of the ovipositor description (usually neglected in Coleoptera taxonomy), which, in the case of this study, proved to be as useful as the aedeagus to differentiate species.

Identifying Dryopidae species used to be almost impossible until recently and is still a difficult task for some genera like *Dryops*, usually requiring the examination of type material. In the case of *Dryops*, there are species very similar to each other externally, and we found that, sometimes, the only structures that can be used to separate them are indeed the genitalia. Despite that, we believe this study will ease studying and determining *Dryops* species in Brazil and help further studies on the Neotropical representatives of the genus. We emphasize that, despite important recent contributions on taxonomy, the family lacks molecular and revisional morphological studies. This includes proper treatment of male and female genitalia as well as a complete taxonomic revision, especially on the most specious and problematic of the Dryopidae genera, like *Pelonomus* and *Dryops*.

## Figures and Tables

**Table 1:** Checklist of Neotropical *Dryops*.

Species	Records
*Dryops aequinocialis Grouvelle, 1896*	Colombia: Magdalena Rive
*Dryops antonioi sp. nov.*	Brazil: states of Paraná and Rio de Janeiro
*Dryops centralis Sharp, 1887*	Panama
*Dryops crassus Grouvelle, 1896*	Brazil: state of Rio de Janeiro
*Dryops cribratus chilensis Hinton, 1939*	Chile
*Dryops cribratus cribratus Grouvelle, 1896*	Brazil
*Dryops detritus (Sharp, 1882)*	Panama: Chiriquí volcano
*Dryops frater Grouvelle, 1902*	Colombia; Venezuela
*Dryops germaini Grouvelle, 1896*	Bolivia: Cochabamba Province
*Dryops gounellei Grouvelle, 1896*	Brazil: state of Minas Gerais
*Dryops hirtus Grouvelle, 1896*	Brazil: state of Rio de Janeiro
*Dryops major Sharp, 1887*	Panama
*Dryops mexicanus (Sharp, 1882)*	Mexico: cities of Jalapa and Cordoba and Tacaná volcano, city of Chiapas
*Dryops musgravei Hinton, 1937*	Brazil: state of Santa Catarina
*Dryops nelsimarae sp. nov.*	Brazil: state of Bahia
*Dryops ovatus Grouvelle, 1890*	Brazil: state of Rio de Janeiro
*Dryops punctipennis (Sharp, 1882)*	Guatemala; Panama: Bugaba District
*Dryops pusillus (Sharp, 1882)*	Mexico; Guatemala: Maria Linda and Naranjo Rivers, and city of San Jerónimo; Nicaragua: Chontales Department; Panama: Bugaba District
*Dryops similis Grouvelle, 1896*	Brazil: states of Bahia and Minas
*Dryops simoneae sp. nov.*	Brazil: state of Rio de Janeiro
*Dryops sobrinus Grouvelle, 1896*	Brazil: states of Bahia and Pernambuco
*Dryops sublaevis Grouvelle, 1896*	Brazil: state of Pernambuco
*Dryops vicinus Grouvelle, 1896*	Brazil: state of Rio de Janeiro

## Data Availability

The original contributions presented in this study are included in the article/Supplementary Material. Further inquiries can be directed to the corresponding author.

## Supplementary Materials

The following supporting information can be downloaded at https://www.mdpi.com/article/10.3390/insects17040430/s1, Table S1: Checklist of Neotropical *Dryops*, with neotropical species and its distributions. Table S2: DwC_Dryops (.xlslx): Table in Darwin Core format with data from collected specimens.
